# A tale of a tail: Structural insights into the conformational properties of the polyglutamine protein ataxin-3

**DOI:** 10.1016/j.ijms.2012.08.032

**Published:** 2013-07-01

**Authors:** Charlotte A. Scarff, Alessandro Sicorello, Ricardo J.L. Tomé, Sandra Macedo-Ribeiro, Alison E. Ashcroft, Sheena E. Radford

**Affiliations:** a Astbury Centre for Structural Molecular Biology, Faculty of Biological Sciences, University of Leeds, Leeds LS2 9JT, UK; b IBMC – Instituto de Biologia Molecular e Celular, Universidade do Porto, Porto, Portugal

**Keywords:** Ataxin-3, PolyQ, Amyloid, Ion mobility spectrometry, Electrospray ionisation-mass spectrometry

## Abstract

Ataxin-3 is the protein responsible for the neurodegenerative polyglutamine disease Spinocerebellar ataxia type 3. Full structural characterisation of ataxin-3 is required to aid in understanding the mechanism of disease. Despite extensive study, little is known about the conformational properties of the full-length protein, in either its non-expanded healthy or expanded pathogenic forms, particularly since its polyglutamine-containing region has denied structural elucidation. In this work, travelling-wave ion mobility spectrometry–mass spectrometry and limited proteolysis have been used to compare the conformational properties of full-length non-expanded ataxin-3 (14Q) and its isolated N-terminal Josephin domain (JD). Limited proteolysis experiments have confirmed that the JD is stable, being extremely resistant to trypsin digestion, with the exception of the α2/α3 hairpin which is flexible and exposed to protease cleavage in solution. The C-terminal region of ataxin-3 which contains the glutamine-rich sequences is largely unstructured, showing little resistance to limited proteolysis. Using ion mobility spectrometry–mass spectrometry we show that ataxin-3 (14Q) adopts a wide range of conformational states *in vitro* conferred by the flexibility of its C-terminal tail and the α2/α3 hairpin of the N-terminal JD. This study highlights how the power of MS-based approaches to protein structural characterisation can be particularly useful when the target protein is aggregation-prone and has intrinsically unordered regions.

## Graphical abstract

**Graphical abstract:**

**Figure:**
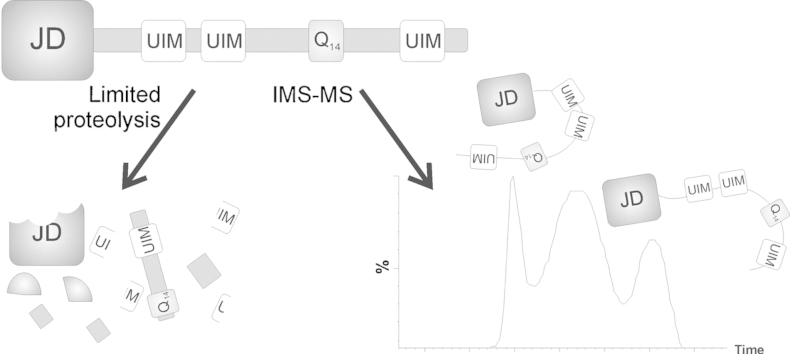

**Highlights:**

► ESI-IMS–MS has been used to characterise ataxin-3 (14Q). ► Ataxin-3 (14Q) shows an array of conformational states. ► The C-terminal domain is largely responsible for its conformational flexibility. ► The dimerisation interface of ataxin-3 is likely to be within the Josephin domain.

## 1 Introduction

### 1.1 Mass spectrometry as a structural biology tool

Over the last 20 years, mass spectrometry (MS) analysis of biological molecules has become an important component of the structural biology toolbox. With the advent of electrospray ionisation (ESI), the ability to ionise and analyse large biological molecules was soon realised and the use of MS in biological applications has become a powerful method of protein structure elucidation, especially for large and complex samples [1,2]. MS analyses benefit from being extremely rapid and having high sensitivity, requiring only picomolar amounts of sample.

Non-covalent MS analyses, where experiments are conducted under carefully controlled solvent and instrumental conditions, can be used to yield information about a protein or protein complex that reflects its behaviour in solution. Consequently, non-covalent MS has been used to mass measure protein complexes ≤10 MDa [3–5] and to deduce their stoichiometry, topological arrangements and dynamics [6–10]. When ESI-MS analysis is coupled with ion mobility spectrometry (IMS) information on analyte shape, including the separation and size determination of co-populated protein conformers, as well as insights into the quaternary structure of macromolecular assemblies, can be gained simultaneously with mass measurements [11–18]. ESI-IMS–MS is particularly useful for the study of heterogeneous samples as it can separate and subsequently identify individual components within a mixture in a single experiment. It can also be used to track biomolecular reactions, yielding information about kinetic intermediates even when these are transient and/or lowly populated [17,19]. Such analyses are of prime importance when NMR spectroscopy or X-ray analysis of the biomolecule, or biomolecular complex, in question is unfeasible or extremely difficult.

Here, ESI-IMS–MS in conjunction with limited proteolysis studies have been used to provide insights into the structure and dynamics of ataxin-3, a clinically important polyglutamine-containing protein.

### 1.2 Ataxin-3 and polyglutamine disease

Ataxin-3 is the protein associated with the neurodegenerative polyglutamine (polyQ) disease spinocerebellar ataxia type 3 or Machado–Joseph disease [20–23]. PolyQ diseases are a group of inherited neurodegenerative disorders, characterised by the accumulation of polyQ-containing neuronal aggregates in particular regions of the brain, whose causative agents are proteins with expanded polyQ stretches [24,25]. These polyQ-expanded proteins are a result of unstable expansion of CAG trinucleotide repeats in their respective gene products, with expansion beyond a particular length, usually >50 contiguous Q residues, resulting in disease [22,26].

An inverse correlation between the number of CAG repeats and the age of onset of disease has been observed for all the polyQ disorders and so polyQ aggregation and polyQ-directed protein misfolding are thought to play key roles in pathogenesis [22,27]. Despite these uniting features of polyQ disease, polyQ proteins show no sequence homology, except in their polyQ tracts, exhibit different functions, result in different pathognomonic symptoms and neurodegenerative profiles, and affect different regions of the brain [22,25,27].

A detailed understanding of the mechanisms by which expression of polyQ-expanded proteins leads to pathology is required in order to generate therapeutic approaches against these disorders [25]. Characterisation of polyQ-containing protein structure, misfolding and aggregation states is an essential component of such studies. PolyQ-containing proteins, however, are particularly challenging to study as they are often structurally dynamic, contain intrinsically disordered regions and are aggregation-prone [22,28]. IMS–MS is therefore an ideal tool for the study of such systems yet, to date, has not been applied rigorously to the structural characterisation of this important set of disease-causing proteins.

### 1.3 Ataxin-3 structure

**Fig. 1:**
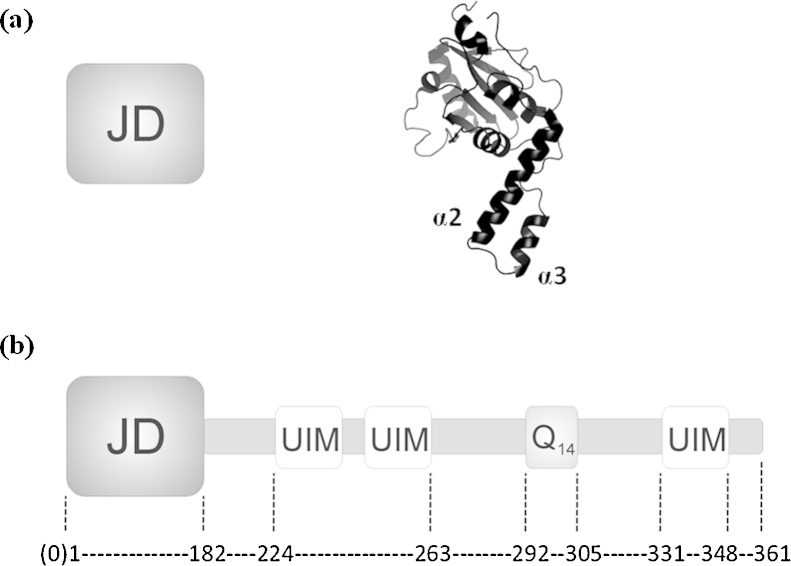
Schematic diagrams of (a) the Josephin domain (JD), and (b) ataxin-3 (14Q). The cartoons illustrate the relative positions of the JD, the ubiquitin interacting motifs (UIMs) and the polyQ stretch (Q_14_). The NMR structure for the isolated JD (PDB 1YZB [29]) with the flexible α2/α3 hairpin is highlighted as a ribbon diagram in (a).

Ataxin-3 is a 42–52 kDa protein that contains an N-terminal Josephin domain (JD; residues 1–182) followed by two ubiquitin interacting motifs (UIMs), a polyQ stretch of variable length, and a variable C-terminal region [29]. The polyQ stretch consists of 12–44 glutamine residues in healthy individuals and results in disease when expanded beyond approximately 54 glutamine residues [30]. In one of the known isoforms of this protein, the predominant isoform in the brain, the C-terminal region includes a third UIM following the polyQ stretch (Fig. 1) [31]. Although the tertiary structure of the JD has been determined by use of solution NMR spectroscopy [29,32,33], little is known about the conformational properties of the full-length ataxin-3 protein, with its C-terminal region herein termed the “tail” [26].

The NMR structure of the JD revealed that this region consists of two sub-domains: a relatively rigid globular catalytic domain and a flexible α2/α3 helical hairpin, the latter which exhibits fast local motions and behaves much as an exposed “waving hand” [29,32–34]. In contrast, the tertiary structure of full-length ataxin-3 has so far evaded detailed structural characterisation at the atomic level. Previous NMR and CD studies on ataxin-3 have suggested that the C-terminal region of ataxin-3 has no defined tertiary structure, yet has multiple secondary structural elements [32]. In addition, NMR data have indicated that the overall fold of the JD is not perturbed by the presence of the C-terminal region of ataxin-3 and that there are no significant interactions between the two regions [32].

To date, there has been a paucity of non-covalent MS experiments on polyQ-containing proteins, including ataxin-3. Indeed, a single ESI-MS study comparing the conformational properties in the presence of organic solvents or heat of the isolated JD with those of a construct of ataxin-3 containing the JD and the adjacent flexible region (183–291) prior to the polyQ stretch has been reported [35], with the authors concluding that the two constructs showed a similar stability to denaturation. Here, by the use of limited proteolysis and ESI-IMS–MS experiments we provide insights into the conformational properties of the non-expanded ataxin-3 (14Q), which contains a stretch of thirteen glutamine residues interrupted by a lysine residue after the first three glutamines, with a single glutamine some six residues later, and by convention is named 14Q, in comparison to the isolated JD. The results provide further evidence that the tail of non-expanded ataxin-3 does not contain defined tertiary structure nor does it interact with the JD. This work demonstrates the power of MS-based approaches to studying polyQ-containing proteins and proteins with intrinsically disordered regions.

## 2 Materials and methods

### 2.1 Protein expression and purification

The cDNAs for human ataxin-3 (14Q) (isoform 2) and the JD (ataxin-3 residues 1–182) were sub-cloned into pDEST17 plasmid vectors [36]. Upstream of the coding site, a sequence coding for an N-terminal hexahistidine tag and a linker region containing a cleavage site for the recombinant tobacco etch virus (rTEV) protease were incorporated. The proteins were expressed in the *E. coli* strain BL21(DE3)-pLysS and purified by nickel-affinity chromatography followed by histidine-tag cleavage with a hexahistidine-tagged rTEV protease [37]. Cleaved recombinant protein was separated from rTEV protease by nickel-affinity chromatography and ataxin-3 (14Q) and JD were then purified to homogeneity by gel-filtration chromatography using a Superdex S200 column. Each recombinant protein construct retains one additional glycine residue at the N-terminus due to the site of rTEV protease cleavage. Herein, this residue is thus numbered zero in the amino acid sequence to avoid confusion.

### 2.2 Sample preparation for MS

Protein samples were buffer exchanged into 10 mM ammonium acetate by use of ZEBA desalting columns (Thermo Fisher Scientific, Loughborough, Leicestershire, UK) and prepared at a concentration of 10–20 μM in 10 mM ammonium acetate for MS analysis. To perform limited proteolysis experiments, bovine trypsin (Sigma Aldrich, Gillingham, Dorset, UK) was added to each protein solution in a 1:100 molar ratio and incubated at 37 °C for different time periods.

### 2.3 ESI-(IMS)–MS analysis

A Synapt HDMS quadrupole time-of-flight mass spectrometer (Micromass UK Ltd., Waters Corpn., Manchester, UK) was used to analyse the JD, ataxin-3 (14Q) and limited proteolysis samples. Samples were introduced into the instrument by direct infusion nanoESI with in-house prepared gold-coated borosilicate glass capillaries. MS and IMS–MS spectra were recorded in nanoESI positive mode using the following instrument parameters: cone voltage 70 V; source temperature 60 °C; backing pressure 3.5 mbar; travelling wave height 9 V; travelling wave speed 350 m/s; IMS gas flow 20 mL/min. Data were processed by use of MassLynx v4.1 and Driftscope software supplied with the mass spectrometer. Estimated collision cross-sectional areas for different species were calculated by use of a non-covalent protein calibration of beta-lactoglobulin, avidin and concanavalin A, following the protocol described by Bush et al. [12]. Tryptic peptides resulting from limited proteolysis of the JD and ataxin-3 (14Q) were separated by IMS and further characterised by collision induced dissociation (CID) MS/MS sequencing when required.

### 2.4 Modelling

Model structures were generated in PyMOL (DeLano Scientific, San Carlos, CA, USA) by use of PDB structure 1YZB [29] as a starting template. Theoretical collision cross-sectional areas for PDB 1YZB and model structures were calculated by use of the MOBCAL algorithm projection approximation [38] corrected for protein shape using the linear fit to the projected superposition approximation (PSA) [39]. This approximation has been shown to be a better estimate of cross-section than the projection approximation as it takes into account the convex-ness or concave-ness of a protein structure [39].

## 3 Results and discussion

### 3.1 Non-covalent ESI-IMS–MS of the JD and ataxin-3

**Fig. 2:**
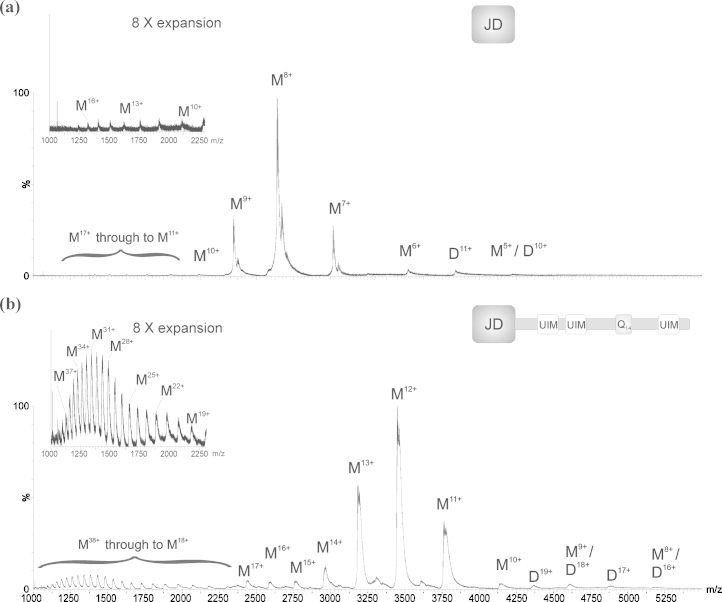
ESI-MS spectra of (a) the isolated Josephin domain (JD) and (b) ataxin-3 (14Q). Insets show the expanded (8×) region *m*/*z* 1000–2500. M, monomer; D, dimer.

The extent of protein ionisation during the ESI process is correlated directly with the surface-exposed area and mass of the protein [40]. The lowest charge states detected within the *m*/*z* spectrum for a protein are most reflective of the native structure of that protein, whilst partially folded protein conformations exhibit intermediate charge states and denatured or unfolded proteins carry the highest number of charges. In the case of the JD, the expected average charge states, based on mass [40], under native conditions and denaturing conditions are 9+ and 22+, respectively. For ataxin-3 (14Q) these are 14+ and 36+. The ESI-MS spectra obtained for the isolated JD (measured mass 21,092.8 Da; calculated mass 21,093.0 Da) and ataxin-3 (14Q) (measured mass 41,309.0 Da; calculated mass 41,307.2 Da) are shown in Fig. 2. One narrow charge state distribution, corresponding to monomeric 6+ to 10+ charge state ions, was populated predominantly in the spectrum of the JD whereas a much broader range of charge states was observed in the spectrum of ataxin-3 (14Q). Both spectra were acquired under identical experimental conditions and comparison of the two spectra thus indicates that the JD adopts mainly a single, compact conformation whereas full-length ataxin-3 (14Q) adopts multiple conformations, some more compact (11+ to 15+) and others more extended (18+ to 38+). The observation of such a wide array of charge states for a protein analysed under native-like solvent conditions, as observed for ataxin-3 (14Q), is unusual but is typical for intrinsically disordered and semi-disordered proteins [41,42]. A second, very minor, charge state distribution (10+ to 17+) was also observed in the JD spectrum indicating the low population of a partially unfolded state (Fig. 2a, inset). It is interesting to note that a population of ions corresponding to a dimeric species was also observed in the spectrum of ataxin-3 (14Q). These were either not observed, or were only very weakly populated, in the spectrum of the isolated JD.

**Fig. 3:**
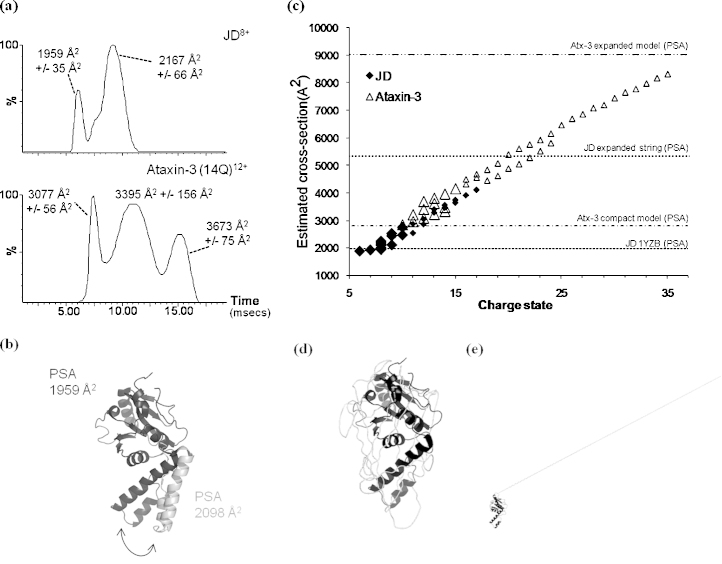
ESI-IMS–MS data for the JD and ataxin-3 (14Q). (a) Extracted arrival time distributions (ATDs) for the most abundant charge states observed within ESI-IMS–MS spectra for the JD (8+) (upper) and ataxin-3 (14Q) (12+) (lower). Estimated cross-sections for each of the main peaks observed in the ATDs, corresponding to maxima drift time values and their approximate dispersion values (based on peak width at half-height), are indicated (b). Schematic of PDB structure 1YZB (black) and modelled structure with α2/α3 hairpin swung out (grey) overlaid; the calculated collision cross-sectional areas for these structures are 1959 and 2098 Å^2^, respectively. (c) ESI-IMS–MS estimated collision cross-sectional areas for all charge states observed for the isolated JD and ataxin-3 (14Q) (black diamond = JD, white triangle = ataxin-3 (14Q)). The size of symbols corresponds to the relative intensities of charge states observed within ESI-MS spectra. Theoretical calculations of cross-sectional areas from model structures are shown as horizontal dotted lines; PSA, projected superposition approximation [39]. Schematics illustrating (d) a compact model and (e) an expanded model of ataxin-3 (14Q).

ESI-IMS–MS experiments on ataxin-3 (14Q) support the notion that this protein adopts a range of compact and partially compact conformations that are co-populated in solution. Extracted arrival time distributions (ATDs) for the dominant charge states observed (in each case the maximum of the lowest charge state distribution which most reflects the native structure) for both the JD (8+) and ataxin-3 (14Q) (12+) exhibited multiple peaks over a range of drift times (Fig. 3a). Two main peaks (one with a shoulder) were observed in the extracted ATD for the dominant charge state of the JD with drift times of 6.16 and 9.23 ms (peak maxima values), from which collision cross-sectional areas of 1959 ± 35 Å^2^ and 2167 ± 66 Å^2^, respectively, were estimated (Fig. 3a). These observations indicated the presence of at least two conformational families of the JD, differing in collision cross-sectional area by approximately 10%. The collision cross-sectional area calculated for the smaller of these two peaks is consistent with the theoretical value for the PDB structure 1YZB (1959 Å^2^). The collision cross-sectional area calculated for the second, more intense peak within the ATD is comparable with that which may be expected for the JD with its α2/α3 hairpin swung out (2098 Å^2^), as modelled in Fig. 3b. The α2/α3 hairpin of the JD domain is known to be solvent exposed and flexible in solution and to behave as an exposed “waving hand”, exhibiting fast local motions [29], and the ESI-IMS–MS results presented here are entirely consistent with this conclusion. In comparison, the broader array of conformations sampled by ataxin-3 (14Q) for the 12+ charge state ions differed in collision cross-sectional area by up to 20% (3077 ± 56 Å^2^, 3395 ± 156 Å^2^, and 3673 ± 75 Å^2^), Fig. 3a. The additional variation in conformational size exhibited by ataxin-3 (14Q) is likely to correspond to additional conformations adopted through movement and flexibility within the “tail”.

Ataxin-3 (14Q) species with estimated collision cross-sectional areas corresponding to a compact model where the protein exhibits a globular conformation (Fig. 3c and d), were observed for the lowest charge states populated, which are most reflective of solution-phase structures. For higher charge states, representative of more expanded conformers, significantly larger collision cross-sectional areas were estimated from ion mobility measurements. Theoretical cross-sections for a fully extended model of the JD, with its amino acids extended in a linear string, and for ataxin-3 (14Q) with a folded JD but a fully extended C-terminal region (Fig. 3e), are shown in Fig. 3c to illustrate the relative range of cross-sections adopted.

The extent that the ESI-IMS–MS estimated collision cross-sectional areas for the partially folded conformers for the JD and ataxin-3 (14Q) mirror the conformeric populations in solution is debatable. Gas-phase unfolding is driven by Coulombic repulsion forces. At low charge states, the effect of Coulomb repulsion between charges is negligible and attractive-folding forces, such as electrostatic interactions which are heightened in the gas phase, dominate [43,44]. Thus, the lowest charge states observed are most reflective of solution-phase structures. As the charge states increase, the effect of Coulomb repulsion becomes more dominant until at the highest charge states the increased Coulombic repulsion between charges forces the protein to adopt more open conformations [44]. Various intermediate conformations may form due to the interplay between attractive-folding and repulsive-Coulombic interactions [45]. For intrinsically disordered proteins, a scaling of overall size as a function of the net charge per residue has been observed [46].

Therefore, whilst some of the estimated collision cross-sectional areas are likely reflective of partially folded solution-phase structures (*e.g.*, charge states 11+ to 15+ for the JD and charge states 16+ to 24+ for ataxin-3 (14Q), where the presence of multiple conformations indicates some degree of structure) the most expanded conformations may be a result of unfolding in the electrospray process due to Coulombic repulsion (charge states 16+ and 17+ for the JD and charge states > 25+ for ataxin-3 (14Q), where single protein conformations are observed exhibiting a uniform increase in cross-sectional area with increase in charge). It is clear, however, from the comparison between the spectra and collision cross-sectional area ESI-IMS–MS estimates for the JD and ataxin-3 (14Q) that ataxin-3 (14Q) adopts much more open conformations than the JD, strongly suggesting that the tail region of ataxin-3 is much less ordered than the JD as it is less restrained in the gas phase.

### 3.2 Limited proteolysis of the JD and ataxin-3

**Fig. 4:**
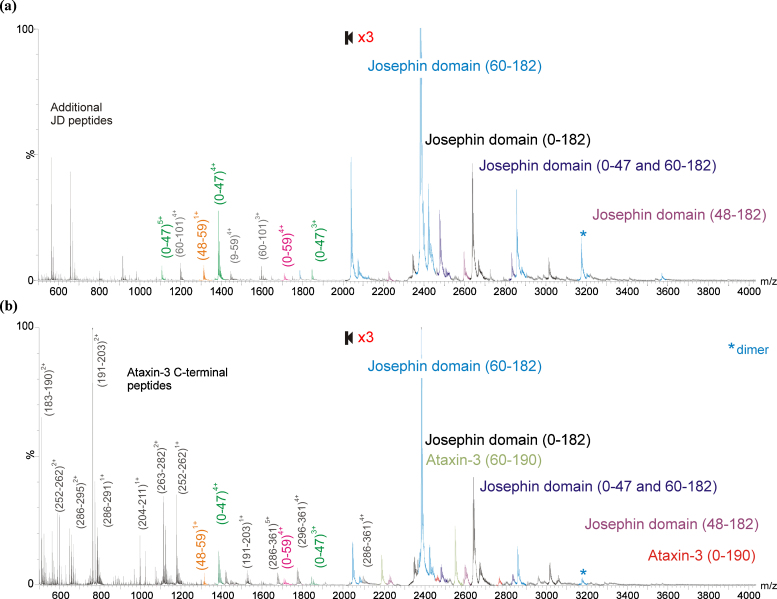
ESI-MS spectra showing the major products of limited proteolysis for (a) the JD, and (b) ataxin-3 (14Q). In each case, spectra were acquired following the incubation of 10 μM protein with 0.1 μM trypsin in 10 mM ammonium acetate, pH 7.4, at 37 °C for 15 min. The protein sequences identified are indicated on the spectra.

**Fig. 5:**
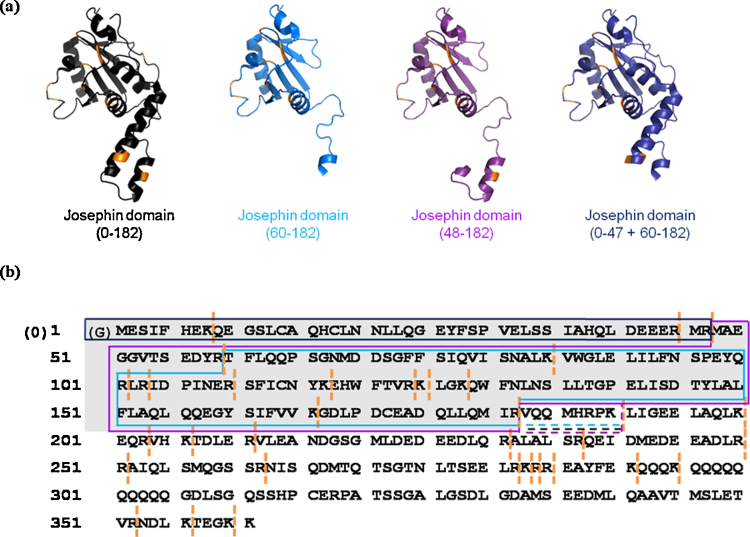
Limited proteolysis of the isolated JD and ataxin-3 (14Q) following incubation of 10 μM protein with 0.1 μM trypsin in 10 mM ammonium acetate, pH 7.4, at 37 °C for 15 min. The major products of limited proteolysis of the JD domain (0–182, 60–182, 48–182, 0–47 + 60–182) are highlighted in (a) on JD structures in the form of ribbon diagrams, taken from the NMR structure for the isolated JD (PDB 1YZB [29]); these products resulted from cleavage at Arg47 and Arg59, both within the α2/α3 hairpin. The amino acid sequence for ataxin-3 (14Q) is shown in (b) with the major products of limited proteolysis (0–182, 60–182, 48–182, 0–47 + 60–182, 0–190 and 48–190). The JD domain (0–182) is highlighted in grey. The trypsin cleavage sites (orange bars) highlighted within the tail region (residues 183–361) all showed evidence of proteolysis. (For interpretation of the references to colour in this figure legend, the reader is referred to the web version of the article.)

Both the JD and ataxin-3 (14Q) were subjected to limited proteolysis using the enzyme bovine trypsin to determine which regions of these proteins are accessible to this protease. Limited proteolysis is a powerful tool that can be used to gain additional structural information regarding a protein's solvent-exposed regions or its domain organisation. ESI-MS spectra obtained following the limited proteolysis of the JD and ataxin-3 (14Q) showed that many of the major products of limited proteolysis are the same for the corresponding regions of both species (Fig. 4). The major products of limited proteolysis observed for the JD are highlighted in Fig. 5a, and a comparison of the products obtained for the JD and the corresponding region of ataxin-3 (14Q) is presented in Table 1.

The key products observed following limited proteolysis of the JD resulted from cleavage of sites within the α2/α3 hairpin (α-helices two and three cover residues 31–47 and 56–62, respectively) at amino acids Arg47 and Arg59. These two cleavage sites are known to be exposed in solution [29] and so proteolysis here was not unexpected. Nonetheless, the unique identification of these two residues as the most exposed sites to trypsin cleavage highlights the power of limited proteolysis to determine conformational properties of a target protein. The core of the JD (residues 60–182) was found to be highly resistant to proteolysis and was still present intact, even after 90 min of exposure to trypsin (data not shown). Interestingly, the experimental conditions were sufficiently gentle that a species corresponding to the 0–47 residue fragment coupled with the 60–182 residue fragment of the JD were observed in the spectra as a non-covalently bound complex; trypsin had cleaved the protein at residues 47 and 59 but the products of cleavage had not dissociated. The individual peptides produced by cleavage, 0–47, 0–59 and 48–59 were also observed (Fig. 4).

In limited proteolysis studies of ataxin-3 (14Q), the N-terminal region (residues 0–182) was found to undergo digestion at a similar rate to the isolated JD and produced the same products as seen for the JD alone (Figs. 4, 5 and Table 1). In addition, tryptic peptides consisting of residues 0–190, 48–190, and 60–190 were observed, resulting from cleavage at Lys190 (Tables 1, 2 and Fig. 5b). The observation of these products indicates that Arg182 (the final residue of the JD within the ataxin-3 (14Q) sequence) is buried, to some extent, within the tertiary structure of ataxin-3 (14Q), limiting access of trypsin to this cleavage site. This is consistent with work by Masino et al. on a similar construct, where equivalent cleavage products were observed for ataxin-3 (14Q) but the isolated JD was not analysed for comparison in that study [26,32]. The generation of the same JD cleavage products in approximately the same intensities upon limited proteolysis of the JD and ataxin-3 (14Q) indicate that there are little or no differences in the accessibility of these cleavage sites in the absence or presence of the C-terminal “tail”. This suggests that the C-terminal region of ataxin-3 (14Q) does not interact with the JD in any significant manner, at least to the extent that the accessibility of Lys or Arg residues within the JD to trypsin cleavage is altered significantly.

Dimeric species of the product 60–182 were observed in the ESI mass spectra following limited proteolysis of both the JD and ataxin-3 (14Q), suggesting that the dimerisation domain of ataxin-3 (14Q) is contained within the core region of the JD. This is consistent with previous studies of ataxin-3 fibril formation, which indicated that protein aggregation initially involves self-association of the JD [36,47], and also with computer predictions which indicated the possibility of three main aggregation-prone regions of ataxin-3 residing within the Josephin domain: namely residues 73–96, 159–167 and 144–154 [35,48].

Peptides resulting from proteolysis at all possible cleavage sites within the ataxin-3 (14Q) C-terminal “tail” (residues 183–361) were identified within ESI-MS spectra after 15 min of trypsin exposure (Figs. 4, 5 and Table 2). The majority of cleavage sites within this region appear to be equally exposed, as peptides resulting from cleavage at these sites are observed instantaneously. This suggests strongly that the C-terminal region of ataxin-3 (14Q) possesses little, if any, persistent tertiary structure that protects its potential cleavage sites from trypsinolysis. Only two species corresponding to the presence of longer products within the C-terminal region, amino acid residues 286–361 and 296–361, were detected. These peptides incorporate the polyQ region in addition to the third UIM. However, there is no trypsin cleavage site between Lys296 and Arg353 within the C-terminal region so further proteolysis within this sequence is necessarily limited, and other peptides were also detected indicating that the trypsin cleavage sites at Arg352 and Lys356 in this region were indeed accessible for proteolysis. Unfortunately, therefore, it is not possible from these data to assess the compactness of the polyQ-containing region of ataxin-3 (14Q). Future work involving the study of ataxin-3 constructs with different proteases and/or polyQ stretches of different lengths will be needed to elucidate the conformational properties of this segment of the polypeptide chain, and its dependence on the polyQ length, in more detail.

## 4 Conclusions

Here we have used ESI-IMS–MS and limited proteolysis experiments to gain new information regarding the conformational properties of a polyQ-containing protein, ataxin-3 (14Q). These experiments show, for the first time, that ataxin-3 (14Q) populates simultaneously a wide range of conformational states in solution, ranging from highly compact to widely extended conformers. Limited proteolysis experiments have indicated that the C-terminal “tail” region of ataxin-3 (14Q) has little resistance to trypsin digestion, consistent with this region being a dynamic, flexible entity able to adopt a wide repertoire of conformational states. However, the observation that the same tryptic peptides arise from the isolated JD and the corresponding JD within ataxin-3 (14Q) following limited trypsin proteolysis suggests that the C-terminal “tail” of ataxin-3 neither interacts with, nor shields, the JD from trypsinolysis. Furthermore, the dimerisation interface of ataxin-3 has been shown to be likely contained within the JD core region encompassing residues 60–182. Together, the results highlight the power of ESI-IMS–MS combined with limited proteolysis to examine the conformational properties of this fascinating class of proteins. Further work, focussing on ataxin-3 variants with glutamine repeats that extend to, and beyond, the pathogenic length, and the interaction of the protein with known modulators of ataxin-3 assembly, will cast new light into how and why these proteins assemble in a polyQ length-dependent manner. The ability of ESI-IMS–MS to analyse complex samples with a range of solution conditions, and to separate and structurally characterise lowly populated species within heterogeneous mixtures, will be essential to unravel the assembly mechanisms of these important protein systems.

## Figures and Tables

**Table 1:** Major products resulting from the limited proteolysis of the Josephin domain (JD) and ataxin-3 (14Q). In each case, ESI-MS spectra were acquired following the incubation of 10 μM protein with 0.1 μM trypsin in 10 mM ammonium acetate, pH 7.4, at 37 °C for 15 min.

Major products of limited proteolysis (amino acid residues)	
Josephin domain	Ataxin-3 (14Q)
60–182	60–182
–	60–190
0–182	0–182
–	0–190
0–47 and 60–182	0–47 and 60–182
–	60–251
48–182	48–182
–	48–190

**Table 2:** Limited proteolysis cleavage products of the C-terminal region of ataxin-3 (14Q) digested with 0.01 (molar ratio concentration) bovine trypsin for 15 min at 37 °C.

Trypsin cleavage site (residue number)	Cleavage product observed	Mass-to-charge ratios of cleavage products observed
190	183–190	1023.6 (1+), 512.28 (2+)
200	191–200	557.3 (2+)
203	191–203	763.9 (2+)
206	191–206	946.0 (2+), 631.0 (3+)
211	204–211	997.5 (1+)
231	212–231	1118.0 (2+), 745.7 (3+)
237	232–237	630.4 (1+)
250	232–250	1102.5 (2+)
251	238–251	874.9 (2+)
262	252–262	1177.6 (1+), 589.3 (2+)
282	263–282	1113.0 (2+), 742.4 (3+)
283	263–283	1177.0 (2+)
284	263–284	837.0 (3+)
291	286–291	786.4 (1+)
295	286–295	649.8 (2+)
295	292–295	531.3 (1+)
352	353–361	1032.6 (1+)
356	357–361	562.3 (1+)
360	–	–
